# Advanced statistical approaches for predicting pain after pediatric thoracotomy: a cross-sectional study using zero-inflated and Poisson models

**DOI:** 10.1186/s44158-024-00188-w

**Published:** 2024-08-05

**Authors:** Alessandro Vittori, Marco Cascella, Piergiacomo Di Gennaro, Giuliano Marchetti, Elisa Francia, Ilaria Mascilini, Riccardo Tarquini, Massimo Antonio Innamorato, Emiliano Petrucci, Franco Marinangeli, Sergio Coluccia, Sergio Giuseppe Picardo

**Affiliations:** 1 Department of Anesthesia and Critical Care, ARCO ROMA, Ospedale Pediatrico Bambino Gesù IRCCS, 00165 Rome, Italy; 2 Department of Medicine, Surgery, and Dentistry, Unit of Anesthesiology, Intensive Care Medicine, and Pain Medicine, University of Salerno, Baronissi, 84081 Salerno, Italy; 3 Epidemiology and Biostatistics Unit, Istituto Nazionale Tumori-IRCCS, Fondazione Pascale, 80100 Naples, Italy; 4 Surgery Unit, Bios Medical Center, 00197 Rome, Italy; 5 Pain Unit, Department of Neuroscience, Santa Maria Delle Croci Hospital, AUSL Romagna, 48121 Ravenna, Italy; 6 Department of Anesthesia and Intensive Care Unit, San Salvatore Academic Hospital of L’Aquila, 67100 L’Aquila, Coppito Italy; 7 Department of Anesthesiology, Intensive Care and Pain Treatment, University of L’Aquila, 67100 L’Aquila, Coppito Italy

**Keywords:** Pain, Pediatric pain, Acute pain, Chronic pain, Thoracotomy, Opioid, Perioperative pain, Pain management, Children, Anesthesia

## Abstract

**Background:**

Thoracotomy is one of the surgical procedures most burdened by chronic post-operative pain. There is poor evidence regarding the possibility that even in pediatric patients, thoracotomy can be followed by post-operative pain. The primary objective of this analysis is to identify associations with home pain therapy, pain intensity, and possible protective factors acting on chronic pain in this population.

**Methods:**

A retrospective cross-sectional study was conducted at Ospedale Pediatrico Bambino Gesù IRCCS. The study included pediatric patients undergoing thoracotomy. For statistical analyses, a logistic model and a zero-inflated strategy were implemented to explore associations and predict factors related to home-based analgesic therapy and pain intensity.

**Results:**

Gender and age were identified as significant factors in the assignment of home therapy, with males having over seven times the risk compared to females (OR = 7.06, 95% CI = [2.11, 29.7]). At the last measurement, pain intensity was positively associated with age and the number of pain events during the week.

**Conclusions:**

The study highlights significant factors influencing post-thoracotomy pain management in pediatric patients. These findings underscore the importance of tailored pain management strategies that consider gender and age to improve post-operative care and outcomes in pediatric thoracotomy patients.

## Background

Chronic pain poses a significant challenge in pediatric healthcare, carrying substantial social and economic implications [1]. The understanding of its origins and effective treatments in pediatric patients is notably limited, making it a complex issue in both clinical practice and research [2, 3]. Importantly, extrapolating scientific evidence from adults to pediatrics is not straightforward [4]. While there is established evidence indicating certain surgical procedures as potential triggers for chronic pain in adults, these findings require validation in the pediatric population [5]. For instance, the association between thoracotomy and chronic post-surgical pain, observed in adults, lacks supporting evidence in pediatric patients [6, 7].

In a prior study, we found no correlation between thoracotomy and chronic pain in children [8]. Despite this, the topic warrants further investigation, especially considering the frequency of thoracotomies, even for benign conditions, in tertiary pediatric hospitals.

Given that chronic post-surgical pain offers an avenue for preventive intervention, understanding its causes becomes crucial from both a research and clinical perspective [9]. In this secondary analysis, we conducted an ensemble of statistical techniques to unravel potential factors related to the home therapy assignment and postoperative pain in pediatric patients undergoing thoracotomy. Additionally, we explored secondary objectives, including variables influencing the persistence of perioperative pain until discharge and the identification of potential shortcomings in the management of acute perioperative pain.

## Methods

### Ethics

The study was approved by the Ethics Committee of the Ospedale Pediatrico Bambino Gesù IRCCS (Ethics Committee n° 957/RA; Chairperson Prof. G. Andria) on 12 December 2013; all study participants, or their legal guardians, signed the informed consent. All methods were performed following the ethical standards as laid down in the Declaration of Helsinki and its later amendments or comparable ethical standards.

### Data source

We considered data collected from pediatric patients undergoing thoracotomy. In the primary analysis, 56 patients were included [7]. For this secondary analysis, additional records were added to the dataset used to better verify the primary outcome. The final dataset was registered on Zenodo [10].

### Data processing

The variables considered for the analyses included age (calculated as the time in months between the intervention date and the date of birth), gender (M = male; F = female), type of intervention (type A = thoracic procedure for pulmonary diseases; type B = Nuss procedures; it is a complex procedure for treating pectus excavatum), anesthesia type (AG = general anesthesia; Aglr = general anesthesia combined with locoregional anesthesia or neuraxial anesthesia), postoperative analgesia (morphine intravenous, peridural analgesia, or acetaminophen at the demand), and peaks at 6-time intervals (T0 = the day of the procedure; T1 = the first day after the procedure; T2 = the second day after surgery; T3 = the third day after the procedure; T4 = the fourth day after the procedure; T5 = the day of discharge from the hospital). Additionally, the highest pain intensity in the week (i.e., week peak) and the number of pain events in the week were computed.

The pain assessment utilizes three different scales: the Numeric Rate Scale (NRS), the Face, Legs, Activity, Cry, Consolability (FLACC) scale, and the Crying Requires Oxygen Increased Vital Signs Expression Sleep (CRIES) scale. The CRIES scale is applicable for infants over 32 weeks of age, the FLACC scale for children over 30 days and under 4 years old, and the NRS scales are used for patients aged 4 years and above. For our analysis, the scoring for all three scales was standardized to reflect the severity of pain. Therefore, a score of 0 indicates no pain, scores ranging from 1 to 3 signify mild pain and are assigned 1 point, scores from 4 to 6 indicate moderate pain and are assigned 2 points, and scores from 7 to 10 reflect severe pain and are assigned 3 points. This method ensures a consistent and comparable assessment of pain across different age groups and pain scales.

### Statistics

A time-based analysis was carried out using the Kruskal–Wallis test [11] and the chi-square test was employed to ascertain whether there was a correlation between the overall variation in the pain scale (Kruskal) or the presence of pain (chi-square) through visits. Subsequently, a significance test was conducted by the estimation of the Intraclass Correlation Coefficient (ICC) [12] to establish whether a mixed-effects model would be more appropriate to describe the phenomena of interest [13].

A univariate analysis was conducted to describe the sample and initially determine possible raw associations between all the covariates with the dependent variables. In particular, chi-square tests and Fisher’s exact tests were adopted for contingency tables analysis, the Wilcoxon-rank-sum test for assessing mean differences by group, and a correlation test (Pearson and Spearman) was assessed. A multivariable analysis was conducted to describe the associations between the two dependent variables and the aforementioned explanatory variables, adjusted for possible confounders. Specifically, a multivariable weighted logistic model was implemented to describe the main associations with the assignment of home pain therapy. Additionally, a zero-inflated model (Regression Models for Count Data in R [rdrr.io]) was initially hypothesized compared to a count model using the Vuong test [14] and then implemented to model the top-rated pain intensity at T4, i.e., the peak pain of the last measurement when the child was still in the hospital. This variable takes on 85.5% of values equal to 0.

The data were analyzed using the R software version 4.2.3 (R Core Teams, R Foundation for Statistical Computing, Vienna, Austria). The toolkit included car, purr, boot, snow, misty, and naniar. Graphical packages were adopted for the visualization of the plots. The graphics packages included ggplot2.

### Study aims

Target variables were detected to characterize the main objectives of this work namely the assignment of home pain therapy (binary: assigned/not assigned) and the highest pain intensity recorded at the last measurement (T4—count variable with many zeros).

The secondary goals of the study encompassed examining various factors that impact the duration of perioperative pain until the point of discharge. This involves a detailed analysis of variables that contribute to the sustained experience of pain postoperatively. Additionally, another secondary objective was to pinpoint potential inadequacies in the current approaches to managing acute perioperative pain. This included a comprehensive evaluation of existing protocols and practices to identify areas where improvements may be necessary.

## Results

Seventy-four children were considered for the analysis. Of these, 68.9% were male, with a mean age of 37.2 ± 52 months. Sixty-nine of them underwent type A intervention (*n* = 87.8%), 7 children underwent type B (9.5%), 2 to type C (2.7%); 67% of the sample received general anesthesia, and 78.4% were given intravenous morphine.

**Fig. 1:**
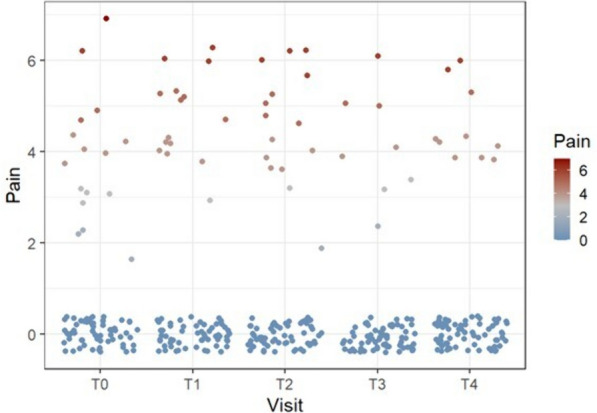
Scatter plot of the pain values at t0 to t4. Legend: no significant variation over time was observed. No significant variation over time was observed

Concerning postsurgical pain, the week peak was 2.0 ± 2.4 NRS units, and most of the patients (93.2%) reported less than 4 non-null pain events in the week (Fig. 1). Further details about the pain events are described in Table 1.

**Table 1:** Main patients’ characteristics

Characteristic	*N* = 74
Gender	
Female	23 (31.1%)
Male	51 (68.9%)
Age (months)	
Mean (SD)	37.2 (52.0)
Median (IQR)	8.3 (3.6, 45.6)
Type of intervention	
A	65 (87.8%)
B	9 (12.2%)
Anesthesia	
Ag	50 (67.6%)
Aglr	24 (32.4%)
Postoperative analgesic	
Morphine iv	58 (78.4%)
Peridural	16 (21.6%)
Home pain therapy	11 (14.9%)
Not assigned	63 (85.1%)
Assigned	11 (14.9%)
Number of pain events/week	
≤ 3	69 (93.2%)
> 3	5 (6.8%)
Week peak	
Mean (SD)	2.0 (2.4)
Median (IQR)	0.0 (0.0, 4.0)
Highest pain intensity T0	
0	58 (78.4%)
2	3 (4.1%)
3	4 (5.4%)
4	5 (6.8%)
5	2 (2.7%)
6	1 (1.4%)
7	1 (1.4%)
Highest pain intensity T1	
0	59 (79.7%)
3	1 (1.4%)
4	6 (8.1%)
5	5 (6.8%)
6	3 (4.1%)
Highest pain intensity T2	
0	59 (79.7%)
2	1 (1.4%)
*3*	1 (1.4%)
*4*	5 (6.8%)
*5*	4 (5.4%)
*6*	4 (5.4%)
Highest pain intensity T3	
*0*	66 (89.2%)
*2*	1 (1.4%)
*3*	2 (2.7%)
*4*	2 (2.7%)
*5*	2 (2.7%)
*6*	1 (1.4%)
Highest pain intensity T4	
0	64 (86.5%)
4	7 (9.5%)
5	1 (1.4%)
6	2 (2.7%)

*Abbreviations*: *GA* general anesthesia, *GA* + *LRA* general anesthesia plus locoregional anesthesia. *Type A surgery* congenital pathology of the lung, *Type B intervention* congenital pathologies of the thoracic cage

The analysis shows that the observation times are not associated with particular pain situations; the Kruskal–Wallis test (*p* = 0.92) and the chi-square test (*p* = 0.29) did not report any association between the pain scale and the observation times. The findings suggest that there is no specific visit associated with a particular variation or trend in the reference pain scale. Moreover, the estimated ICC was 0.27, which was statistically not higher than the predetermined minimum value for an influence of the NRS pain trend over the 5 available times (*F*-test *p* = 0.69). Therefore, since the behavior of the single case did not follow a well-defined pattern through the follow-up visits in the 5 measurements, the mixed-effects methodology was not performed. The time-independent structure was retained, although the pain peak and the number of events in the week were considered exogenous variables for the models.

Referring to home pain therapy (Table 2), the univariable analysis (Table 2, left side) showed that gender was not found significant such as the type of intervention, anesthesia, and postoperative analgesic therapy.

**Table 2:** Univariable and multivariable analyses results for home pain therapy assignment

Characteristic	*N*	Univariable analysis^a^			Multivariable analysis		
		Home pain therapy			Assigned vs not assigned		
		Not assigned (*N* = 63)	Assigned (*N* = 11)	*p* value^‡^	OR	95% CI	*p* value ^§^
(Intercept)	74				0.01	0.00, 0.10	** < 0.001**
Gender				0.156			** < 0.001**
Female	23	22 (34.9%)	1 (9.1%)		—	—	
Male	51	41 (65.1%)	10 (90.9%)		7.06	2.11, 29.7	
Age	74	33.4 (47.5)	59.2 (71.6)	**0.028**	0.94	0.89, 0.98	**0.005**
$\sqrt{\text{Age}}$	74				2.17	1.25, 4.00	**0.005**
Type of intervention				0.125			0.220
A	65	57 (90.5%)	8 (72.7%)		—	—	
B	9	6 (9.5%)	3 (27.3%)		3.51	0.48, 34.7	
Anesthesia				> 0.999			0.397
Ag	50	42 (66.7%)	8 (72.7%)		—	—	
Aglr	24	21 (33.3%)	3 (27.3%)		1.85	0.44, 8.05	
Postoperative analgesic				0.694			0.822
Morphine iv	58	50 (79.4%)	8 (72.7%)		—	—	
Peridural	16	13 (20.6%)	3 (27.3%)		1.17	0.30, 4.81	
Week peak	74	1.8 (2.4)	3.3 (2.2)	0.069	1.22	0.99, 1.52	0.064
Number of pain events/week				**0.021**			**0.002**
≤ 3	69	61 (96.8%)	8 (72.7%)		—	—	
> 3	5	2 (3.2%)	3 (27.3%)		27.4	2.87, 604	

*Abbreviations*: *GA* general anesthesia, *GA* + *LRA* general anesthesia plus locoregional anesthesia. *Type A surgery* congenital pathology of the lung, *Type B surgery* congenital pathologies of the thoracic cage

^a^For numerical variables means (SDs) were reported

^‡^Fisher’s exact test for categorical variables, Wilcoxon rank sum test for numerical variables. °Calculated in months

^§^*z*-test

A borderline association was found for the week peak (3.3 vs 1.8 NRS units), although it did not reach the significance level (*p* = 0.07). On the other side, the number of pain events in the week was significant; children with home pain therapy had a 27% probability of having more than 3 peaks per week, compared to 3% for those without (*p* = 0.02). Moreover, those who received home pain therapy were found meanly older (59 vs 33 months, *p* = 0.03).

**Fig. 2:**
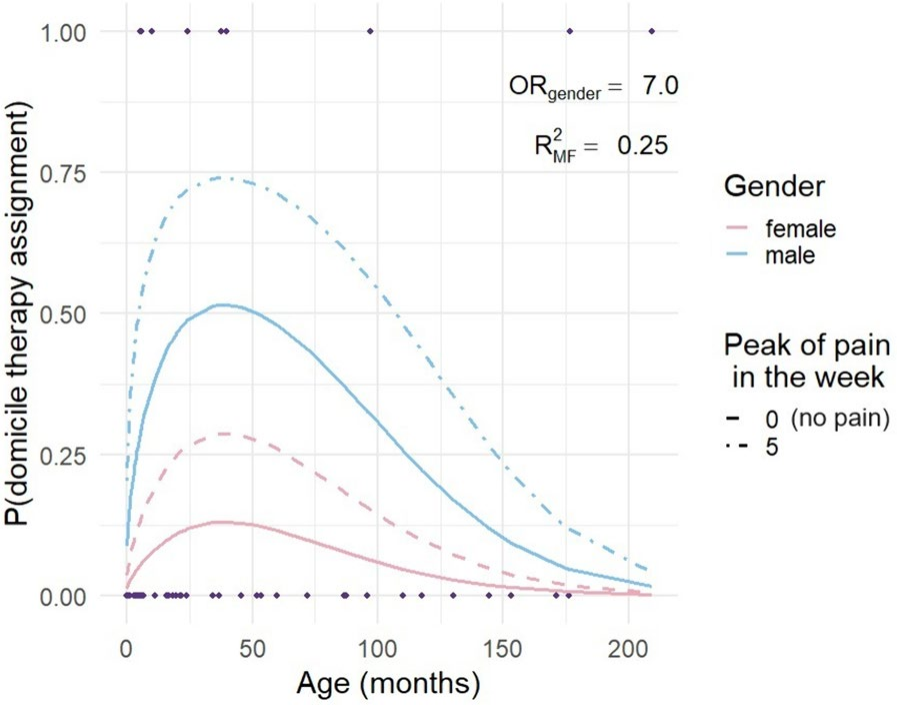
Multivariable logistic regression model. Legend: probability of undergoing domicile therapies, focus on gender (OR = 7.02, *p* < 0.01) and pain peek in the week (OR = 1.22, *p* = 0.06). McFadden R-squared as 0.25. Probability of undergoing domicile therapies, focus on gender (OR = 7.02, *p* < 0.01) and pain peek in the week (OR = 1.22, *p* = 0.06). Mc Fadden *R*-squared as 0.25

The multivariable analysis, (Table 2 right side), showed a significant association with gender. Interestingly, male children were 7 times more likely to receive home pain therapy (OR = 7.06, 95% CI = [2.11, 29.7]). Additionally, age was inversely associated although the relationship with home pain therapy had a sublinear component (Fig. 2), wherein for younger ages, the probability (*P* on the *y*-axis) of home pain therapy first increased and then decreased. In this context, an association of home pain therapy seemed to be strongly positive for younger patients and then progressively was reduced as the age increased (following a parabolic shape, see Fig. 2). Figure 2 focuses on gender analysis and highlights the above findings (solid lines). The relationships between the home pain therapy assignment and the week peak were indicated; the dashed lines illustrate whether the child had no episodes of pain (intensity = 0 always and peak = 0) or if he/she reached the peak of five through the 5 measurements (t0, t4). Although the association was borderline (*p* = 0.06), it was found that for each additional unit on the NRS scale a 22% higher probability of being assigned home pain therapy. Regarding the number of pain events per week, children who experienced more than three painful events had 27 times greater odds of getting home pain therapy prescribed (*p* < 0.01).

**Fig. 3:**
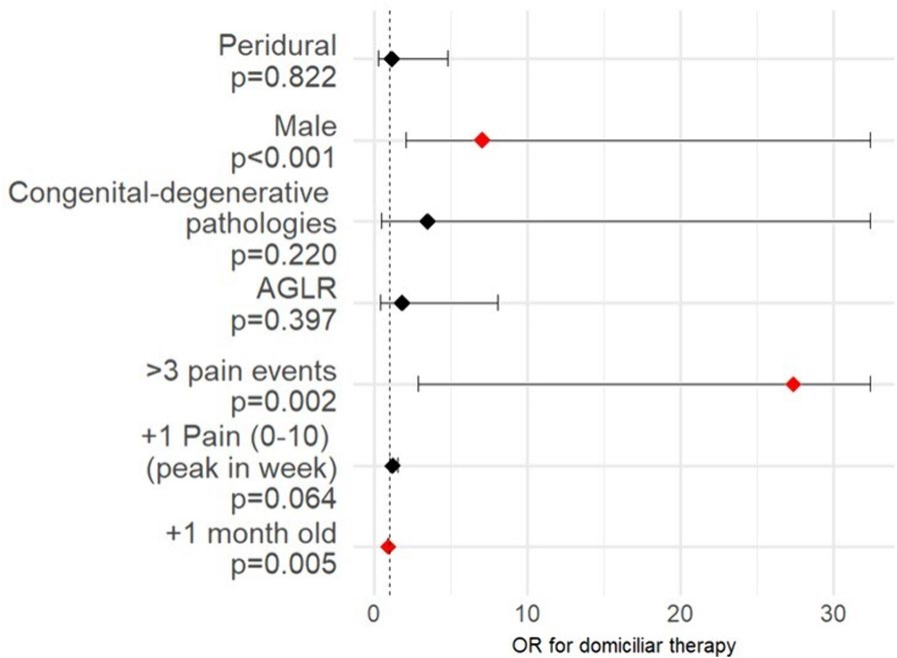
Forest plot of the logistic multivariate model. Legend: pain events are considered within a week

In Fig. 3, model results (ORs) were plotted depicting the considered variables association strength.

**Fig. 4:**
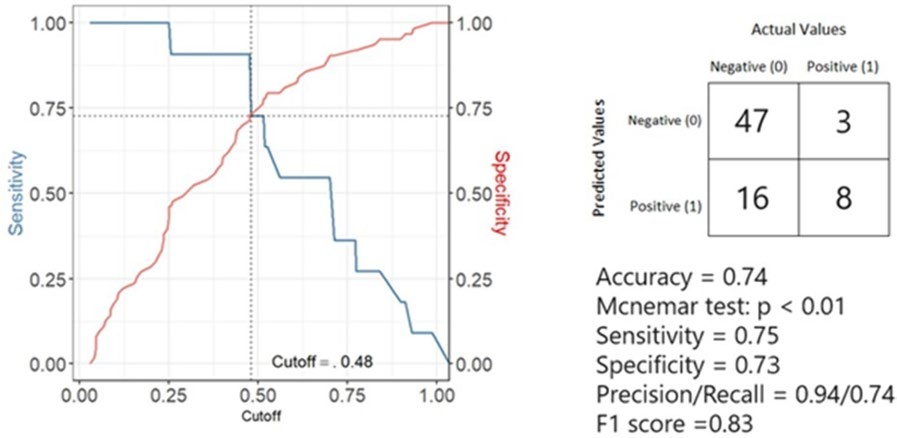
Performances. Legend: best cut-off for achieving the best performance of the model and performance statistics applied after the cut-off

To verify the best performance of the obtained weighted model (Table 2, right; Fig. 2), the optimal cut-off related to the probability of home pain therapy assignment was calculated based on the compromise between model results in terms of specificity and sensitivity. The best-calculated cut-off was found as 0.48 (Fig. 4). This means that, although initially there was a 49% probability that a child would not be identified as needing home pain therapy, with this new specification, there was a more accurate estimate of this risk.

Regarding the highest pain intensity recorded at T4, the results are shown in Table 3.

**Table 3:** Univariable analysis and multivariable zero-inflated model for domicile pain intensity

	Univariable analysis			Multivariable analysis					
				Count part (Poisson link)			Zero-inflated part (logit link)		
Characteristic	*N*	Mean (SD)/ρ^b^	*p* value^2^	OR	95% CI	*p* value^3^	OR	95% CI	*p* value^3^
Maximum recorded pain at T4 (dependent variable)									
0	64								
4	1								
5	2								
6	7								
Gender			0.961			0.054			0.067
Female	23	0.609 (1.64)		—	—		—	—	
Male	51	0.61 (1.56)		0.42	0.17, 1.02		0.02	0.0, 1.34	
Age	74	0.49	** < 0.001**	1.01	0.96, 1.06	0.78	1.14	0.93, 1.39	0.199
$\sqrt{\text{Age}}$	74	0.5	** < 0.001**	0.75	0.27, 2.08	0.586	0.02	0.0, 1.39	0.071
Type of surgery			** < 0.001**			0.058			**0.046**
A	65	0.43 (1.39)							
B	9	1.89 (2.26)		5.23	0.94, 29.71		1122.8	—	
Anesthesia			0.466	^a^			^a^		
GA	50	0.66 (1.53)							
GA + LRA	24	0.50 (1.69)							
Postoperative analgesic			0.973	^a^			^a^		
Morphine ev	58	0.57 (1.44)							
Peridural	16	0.75 (2.04)							
Max intensity/week	74	0.45	** < 0.001**	1.74	1.40, 2.14	** < 0.001**	3.52	0.59,21.06	0.168
Number of pain events/week			**0.002**	^a^			^a^		
≤ 3	69	0.45 (1.37)							
> 3	5	2.80 (2.68)		1.04					

^a^Not included in the model

^b^Mean (sd) or correlation index ρ (by Pearson) were reported if the strata variable was categorical or numerical

^2^wilcoxon-rank-sum test and null-correlation test if strata variable was categorical or numerical

^3^*z*-test; all tests were conducted at the 95% confidence level

Sixty-four out of seventy-four patients (84.6%) did not report pain at time T4. Among those who did report pain, 1 patient had a pain score of 4, 2 patients had a score of 5, and 7 patients (9.5%) had a score of 6. Pain intensity was positively highly correlated with age (*p* = 0.49, *p* < 0.01). Type B interventions were generally associated with greater pain (1.9 vs 0.4 mean intensity, *p* < 0.01). As expected, T4 intensity pain was positively related to the maximum pain intensity recorded during the week (*ρ* = 0.45, *p* < 0.01), and with the number of pain events: the more events were recorded, the more severe the pain at t4 is (2.8 vs 0.4, *p* < 0.01).

The Vuong statistic was *z* = 4.04 (*p* < 0.01); therefore, the ZIP model was confirmed as better to capture the zero-inflated phenomenon.

In analyzing the zero-inflated logit estimations, although not reaching significance, males were more likely to suffer more in almost all cases (OR = 0.02, *p* = 0.07). The type of intervention B was inversely associated with the probability of experiencing pain events (*p* = 0.05), even though the estimates for this coefficient were extremally distorted. In the end, there doesn't seem to be an association between the pain event at time T4 and the week peak (*p* = 0.17).

In the count model (Poisson), in the same way, although not reaching significance, males seem to have 58% less NRS score compared to females. It appeared that male children were more likely to have events during the week but of less intensity. Furthermore, not significant but borderline is the type of intervention. Children undergoing intervention of type “B” were on average 5 times more likely to have increased pain by NRS score measurement (OR = 5.2, *p* = 0.06). Then, the number of pain events per week was found significant: each additional pain event in the week led to a 74% increase in undergoing a higher NRS score (*p* < 0.01).

## Discussion

In this secondary analysis, results from our referral study were confirmed, and the association between thoracotomy and 1-week postoperative pain in pediatric patients was not demonstrated. This finding is crucial since there is limited evidence regarding factors linked to post-thoracotomy chronic pain in children [15]. Therefore, given that post-surgical chronic pain is a type of chronic pain that can be prevented, our statistical investigations on associations with different variables linked to the management of acute perioperative pain post-thoracotomy can be of great interest [9, 16].

Notably, the results highlighted a significant gender difference: male children were found to be seven times more likely to receive home pain therapy compared to female children (OR = 7.06, 95% CI = [2.11, 29.7]). Nevertheless, the study population included a majority of males (68.9%) with a mean age of 37.2 ± 52 months. The Nuss procedure (type B intervention) is particularly known for its complexity and associated high pain levels (in our analysis, 1.9 vs 0.4 mean intensity, *p* < 0.01). This procedure is primarily performed to treat pectus excavatum, a condition that predominantly affects males and often requires surgical correction during adolescence. Given the higher prevalence of males undergoing type B interventions, it is plausible that this subgroup contributes significantly to the observed gender difference in home pain therapy assignment. Further studies are needed to dissect this issue.

Furthermore, it emerged that there was no specific postoperative day or examination that was characterized by pain requiring a rescue analgesic dose. The data suggested that within perioperative pain management protocols, there were no inherent structural flaws, such as, for instance, insufficient analgesic therapy during physiotherapy. However, observed variations were presumed to be attributed to individual variables. It is crucial to note that, especially in children, the customization of analgesic therapy is essential, given the influence of additional variables. Factors such as parents’ catastrophic thinking and other psychological considerations underscore the need for a personalized approach to analgesic strategies, a consideration even more pronounced in pediatric cases compared to adults [17].

Additionally, the need to take rescue doses of analgesics is associated with a high probability of being discharged from the hospital with ongoing analgesic therapy. This result is of paramount importance as it can be interpreted both as correct pain management, in full adherence to the “Hospital Without Pain” project, and as a warning bell for acute pain that can transform into chronic pain [18, 19]. It can be hypothesized that the lack of post-thoracotomy chronic pain is also related to proper perioperative pain management [20].

Notably, contrary to what is stated in adult patients, locoregional anesthesia showed no protective role against post-thoracotomy chronic pain in pediatric patients [21]. Our results demonstrated no difference in the prevalence of postoperative pain between patients undergoing surgical procedures under general anesthesia and those undergoing procedures under balanced anesthesia (general and locoregional). These results suggest that deeper investigations are needed to dissect the role of locoregional anesthesia in pediatric patients [22]. In this regard, it could be possible that the neuroplasticity of pediatric patients has a protective role that outweighs the surgical technique and the type of anesthesia used [23]. Significantly, younger patients have more pain in the immediate perioperative period, in line with their greater pain perception (inversely proportional to age), and then it decreases over time, precisely due to greater neuroplasticity [24].

On the other hand, patients who experienced more painful events were precisely older in age, and they reported a very strong statistical association with home analgesic therapy. As previously discussed regarding gender prevalence, this finding can be explained by the fact that almost all older patients, usually adolescents, underwent the Nuss procedure, which is one of the most painful among thoracic surgical procedures [25, 26].

Another interesting result is that males were discharged from the hospital with home analgesic therapy much more likely than females, regardless of age. The models highlighted results concerning gender differences. It was shown that male children were more likely to have pain events during the week (see logistic model), but of less intensity (as seen in the ZIP model for T4 measurement), also if this last association was found borderline. This further emphasizes that the pain phenomenon is different from that which is experienced in adults [27, 28], and underscores the necessity to structure dedicated follow-up programs, also utilizing digital technologies and telemedicine tools [29, 30].

## Limitations

Our secondary analysis has some limitations. Firstly, it is a retrospective cross-sectional study. However, most studies on chronic pain are cross-sectional precisely because of the difficulty in following up with patients over time [31]. The second limitation is that it is not a multicenter study. However, the study has a significant sample and investigates the presence of postoperative pain when patients are still in pediatric age. It would be desirable to design large and multicenter studies to better evaluate all the characteristics of chronic pain in pediatric patients. Another key limitation concerns the pain assessments. The final evaluation (the day of discharge) may correlate with the necessity of prescribing home pain therapy but does not necessarily correlate with the onset of chronic pain. To establish such a correlation, a follow-up at 1 month and 3 months post-surgery would be necessary.

## Conclusions

In contrast to adults, pediatric patients undergoing thoracotomy, including adolescents, do not typically experience chronic pain. Nevertheless, thoracotomy remains a notably painful procedure necessitating tailored pain management during the perioperative phase. Notably, the frequency of painful events throughout the recovery process serves as a predictive factor for determining the necessity of discharging the patient with home-based therapy.

## Data Availability

No datasets were generated or analysed during the current study.

## Abbreviations

Ag: General anesthesia
Aglr: General anesthesia combined with locoregional anesthesia
CRIES: Crying Requires Oxygen Increased Vital Signs Expression Sleep
FLACC: Face, Legs, Arms, Cry and Consolability
ICC: Intraclass Correlation Coefficient
NRS: Numerical Rating Scale
ORs: Model results (ORs)
